# Modified Frailty Index as a Predictor of Adverse Outcomes in Elective Primary Hip and Knee Replacement Surgery Patients at a Tertiary Care Hospital in Pakistan: A Cross-Sectional Study

**DOI:** 10.7759/cureus.55783

**Published:** 2024-03-08

**Authors:** Moiz Ali, Mohammad K Safri, Muhammad Abdullah, Fareeha Nisar, Haider A Lakdawala, Manzar Abbas, Riaz H Lakdawala, Shahryar Noordin

**Affiliations:** 1 Orthopedic Surgery, Aga Khan University, Karachi, PAK

**Keywords:** post-operative mortality, post-operative morbidity, total knee replacement, total hip replacement, modified frailty index

## Abstract

Objective

The objective was to evaluate the modified frailty index as a predictor of early (within 30 days) postoperative complications in total joint arthroplasty patients, in a low middle-income country.

Material and methods

A cross-sectional study was carried out which included patients with ages ranging from 23 to 86 years, who underwent elective primary Total Hip or Knee Arthroplasties (TKA or THA) between December 2021 and February 2023. Modified frailty index (mFI-5) was calculated and 30-day morbidity and mortality were recorded. Post-operative complications were categorized as either surgical or medical and recorded.

Results

A total of 175 patients were included, amongst whom the majority were females (68.6%, n=120) and the mean age was 60.5 ± 13.2 years. 85 patients (48.6%) had a mFI-5 score of one while 48 patients (27.4%) had a score of two. Superficial surgical site infection was the most common complication overall in 6 patients (3.4%); however, no case of prosthetic joint infection was noted. Deep vein thrombosis (DVT) was the most common medical complication (1.7%, n=3). 5 patients (2.9%) required re-admission and two mortalities were recorded within the 30-day interval. A significant association was noted between post-operative surgical complications and mFI-5 score (*p*-value = < 0.001), with the risk of complications increasing with a higher mFI-5 score. Smoking was noted to be a risk factor for post-operative medical complications as well as 30-day mortality.

Conclusion

The current study shows that the mFI-5 index can effectively be used as a predictor of postoperative complications in the South Asian region such as Pakistan. This should be calculated routinely and can be used as a tool for pre-operative assessment and counseling.

## Introduction

Osteoarthritis of hip and knee joints is on the rise in developing countries and the demand for surgical management is increasing with the rising incidence [1]. In recent years, Total Hip and Knee Arthroplasties (THA and TKA) have become common across the world and are considered amongst the safest procedures in orthopedic surgery [1,2]. They have also been shown to have a phenomenal impact in improving the overall quality of life [3].

However, these surgeries are still associated with risks of postoperative local as well as systemic complications [4,5],^ ^which can be burdensome for the patients both financially and medically in terms of morbidity [6]. In one of the national database studies conducted in 2017, it was shown that the incidence of major postoperative complications was increased in patients with pre-existing co-morbid conditions, most significant of which were congestive and valvular heart diseases [7].

In previous studies, many risk stratification tools have been used for the assessment and predictability of postoperative complications [8]. Modified frailty index (mFI) has recently been used to evaluate hip and knee arthroplasty surgery patients [9]. Frailty is defined as the "decline of normal body physiology involving multiple organ systems", and the frailty index identifies susceptible patients for complications during the post-surgical period [10]. It was ﬁrst presented by the Canadian Study of Health and Aging (CSHA) and was later modified by Velanovich et al. to an 11-item index, derived from the original 70-item CSHA frailty index [10,11]. Later in 2018, Traven et al. published a five-item modified frailty index (mFI-5), and reported it to be equally effective for predicting postoperative complications [9].

To the best of our knowledge, there is a scarcity of literature regarding risk stratification and prediction of adverse outcomes following Total Joint Arthroplasty (TJA) from our region. Therefore, the objective of this study was to evaluate the modified frailty index as a predictor of early postoperative complications (within 30 days) in knee and hip arthroplasty patients from a developing country.

## Materials and methods

A prospective cross-sectional study was carried out at a tertiary care center in Pakistan in which patients with ages ranging from 23 to 86 years who underwent elective primary TKA or THA for degenerative joint disease between December 2021 and February 2023 were included. Patients with less than 30 days of follow-up, or patients with underlying musculoskeletal tumors undergoing arthroplasty as a limb salvage procedure were excluded. Approval was taken from the Ethical Review Committee at our institution.

All patients undergoing total joint arthroplasty were administered a single dose of anti-coagulation (low molecular weight heparin or heparin if creatinine deranged) the night before surgery, which was then restarted from the first postoperative day and continued until the patient was admitted. This was switched to oral aspirin 75 mg once daily at the time of discharge. Pre-operative antibiotic prophylaxis was given to all patients which included first-generation cephalosporin, and this was continued till the third postoperative day. 

Demographic characteristics including age, gender, and comorbid conditions were recorded for all included patients, and the modified frailty index (mFI-5) was calculated (Table 1) [9]. Post-operative complications were classified as either surgical (local) or medical (systemic) and recorded in the proforma. Surgical complications included superficial surgical site infection (SSSI), prosthetic joint infection (PJI), or implant dislocation, while medical complications included deep vein thrombosis (DVT), pulmonary embolism, myocardial infarction, or cardiopulmonary arrest. Any 30-day readmission or mortality was separately recorded in the proforma.

**Table 1:** Modified frailty index – 5 (mFI-5) criteria

Modified frailty index (5 item) mfi-5	
1	Congestive heart failure
2	Diabetes mellitus (insulin dependent or noninsulin dependent)
3	Chronic obstructive pulmonary disease or pneumonia
4	Dependent functional health status (total or partial) at time of surgery
5	Hypertension requiring medication

SSSI was diagnosed clinically with the presence of any incision site infection involving the skin, subcutaneous, or deep tissues while sparing the joint [12]. PJI, on the other hand, was defined using the Musculoskeletal Infection Society (MSIS) criteria in which PJI is diagnosed when 1) prosthesis is communicating with a sinus tract visible on examination; or 2) positive cultures are obtained from at least two different tissues/fluid samples from within the joint cavity; or 3) four of the following six criteria are present - raised serum erythrocyte sedimentation rate (ESR) and C-reactive protein (CRP), raised synovial fluid white blood cell count or neutrophil percentage, purulent fluid in the joint, isolation of a pathogen in periprosthetic fluid or tissue culture, or more than five neutrophils per high-power field (hpf) in five hpfs observed from analysis of periprosthetic tissue at ×400 magnification [12].

Data was analyzed using SPSS version 23 (IBM Corp., Armonk, USA). Qualitative variables like gender and smoking status were recorded as frequencies and percentages, whereas quantitative variables, e.g., age were recorded as mean ± standard deviation. Chi-square test was used to analyze any association between mFI-5 and other demographics with postoperative complications and a p-value of <0.05 was considered significant.

## Results

A total of 175 patients were included, amongst whom 120 were females (68.6%). Table 2 shows the demographics of these participants. The mean age of all included participants was 60.5 ± 13.2 years while the mean BMI was 31.1 ± 5.3 kg/m2. The mean length of hospital stay was 7.0 ± 1.7 days. All patients who underwent TKA were noted to have simultaneous bilateral procedures, while patients with THA had undergone unilateral surgeries.

**Table 2:** Characteristics and postoperative outcomes of the study population. *ASA - American Society of Anesthesiologists **mFI-5 - modified Frailty Index

Characteristics	Frequency (%)
Gender	
Male	55 (31.4)
Female	120 (68.6)
ASA* Level	
I	16 (9.1)
II	110(62.9)
III	48 (27.4)
IV	1 (0.6)
mFI-5** score	
0	31 (17.7)
1	85 (48.6)
2	48 (27.4)
3	11 (6.3)
4	0
5	0
Smoker	
Yes	15 (8.6)
No	160 (91.4)
Arthroplasty	
Knee	99 (56.6)
Hip	76 (43.4)
Surgical Complications	
Superficial Surgical Site Infection	6 (3.4)
Implant Dislocation	1 (0.6)
Medical Complications	
Pulmonary embolism	1 (0.6)
Myocardial Infarction	1 (0.6)
Deep Vein Thrombosis	3 (1.7)
Cardiopulmonary arrest	1 (0.6)
30-day readmission	
Yes	5 (2.9)
No	170 (97.1)
30-day mortality	
Yes	2 (1.2)
No	173 (98.8)

Patients’ comorbidity was assessed using mFI-5 scores which ranged from 0 to 5. Eighty-five patients (48.6%) had a score of 1 while 48 patients (27.4%) had a score of 2, details of which are provided in Table 1. Smoking status was also taken into consideration for postoperative complications, and 15 (8.6%) of the patients were smokers.

With regards to postoperative complications, surgical complications, medical complications, 30-day readmission, and 30-day mortality were assessed separately. Superficial surgical site infection was noted to be the most common complication overall in six patients (3.4%), whereas no case of PJI was noted. DVT was found to be the most common medical complication. Five patients (2.9%) required re-admission within 30 days postoperatively, whereas two (1.2%) mortalities were recorded within the 30-day interval.

A statistically significant association was noted between the occurrence of postoperative surgical complications and the mFI-5 score (p-value = 0.001), with the risk of complications increasing with a higher mFI-5 score (Table 3). Other factors including American Society of Anesthesiologists (ASA) level, gender, or smoking status were not noted to be significantly associated with postoperative surgical complications. Although whether undergoing THA or TKA was not found to be statistically significant with surgical complications, five out of the six patients with SSSI were those who had undergone TKA, while one of the patients undergoing THA suffered from SSSI and another had an implant dislocation. However, with respect to medical complications, smoking was found to be a significant risk factor (p-value = 0.027) as shown in Table 3, while the mFI-5 score did not reveal a significant association. Age was also not found to have any significant association with the occurrence of either surgical or medical complications (p-value = 0.34 and 0.81, respectively). The type of arthroplasty again did not have a significant association with the occurrence of medical complications. Out of the four complications in the THA group, two had DVT, one had pulmonary embolism, and one had a myocardial infarction. Whereas, in the TKA group, one patient had DVT and one had a sudden cardiopulmonary arrest. 

**Table 3:** Association of baseline characteristics with surgical and medical complications *mFI-5 - modified Frailty Index - 5 ** ASA - American Society of Anesthesiologists

Characteristics	Surgical Complication (%)		P-value	Medical Complication (%)		P-value
	Yes	No		Yes	No	
Gender			0.14			0.32
Male	4 (7.3)	51 (92.7)		3 (5.5)	52 (94.5)	
Female	3 (2.5)	117 (97.5)		3 (2.5)	117 (97.5)	
mFI-5* score			0.001			0.58
0	0	31 (100)		0	31 (100)	
1	3 (3.5)	82 (96.5)		4 (4.7)	81 (95.3)	
2	1 (2.1)	47 (97.9)		2 (4.2)	46 (95.8)	
3	3 (27.3)	8 (72.7)		0	11 (100)	
ASA** Status			0.85			0.30
I	0	16 (100)		0	16 (100)	
II	5 (4.5)	105 (95.5)		6 (5.5)	104 (94.5)	
III	2 (4.2)	46 (95.8)		0	48 (100)	
IV	0	1 (100)		0	1 (100)	
Smoking			0.41			0.027
Yes	0	15 (100)		2 (13.3)	13 (86.7)	
No	7 (4.4)	153 (95.6)		4 (2.5)	156 (97.5)	
Arthroplasty			0.42			0.24
Knee	5 (5.1)	94 (94.9)		2 (2.0)	97 (98.0)	
Hip	2 (2.6)	74 (97.4)		4 (5.3)	72 (94.7)	

All five (2.9%) patients who required re-admission within 30 days postoperatively had undergone THA (p-value = 0.01) and no other factor was noted to be associated with the risk of re-admission as shown in Table 4. With regards to 30-day mortality, smoking was again noted to be a significant risk factor (p-value = 0.035) (Table 4).

**Table 4:** Association of baseline characteristics with 30-day re-admission and mortality *mFI-5 - modified Frailty Index - 5 **ASA - American Society of Anesthesiologists

Characteristics	30-day re-admission (%)		P-value	30-day mortality (%)		P-value
	Yes	No		Yes	No	
Gender			0.68			0.34
Male	2 (3.6)	53 (96.4)		0	55 (100)	
Female	3 (2.5)	117 (97.5)		2 (1.7)	118 (98.3)	
mFI-5* score			0.44			0.54
0	0	31 (100)		0	31 (100)	
1	3 (3.5)	82 (96.5)		2 (2.4)	83 (97.6)	
2	1 (2.1)	47 (97.9)		0	48 (100)	
3	1 (9.1)	10 (90.9)		0	11 (100)	
ASA** Status			0.84			0.75
I	0	16 (100)		0	16 (100)	
II	4 (3.6)	106 (96.4)		2 (1.8)	108 (98.2)	
III	1 (2.1)	47 (97.9)		0	48 (100)	
IV	0	1 (100)		0	1 (100)	
Smoking			0.35			0.035
Yes	1 (6.7)	14 (93.3)		1 (6.7)	14 (93.3)	
No	4 (2.5)	156 (97.5)		1 (0.6)	159 (99.4)	
Arthroplasty			0.01			0.85
Knee	0	99 (100)		1 (1.0)	98 (99)	
Hip	5 (6.6)	71 (93.4)		1 (1.3)	75 (98.7)	

## Discussion

With recent advances in surgical techniques and medical management options, total joint arthroplasty, including hip and knee replacements, is considered one of the safest procedures in orthopedics [1,2]. However, they still entail a major undertaking on behalf of the patient and are associated with major surgical as well as systemic complications [13]. Some of the common complications reported in the literature include surgical site infections, deep vein thromboses, pulmonary embolism, and pneumonia [13-15].

In accordance with previously reported studies, the most common complication that we encountered was SSSI in six (3.4%) of our patients, all of whom were managed with local wound care and antibiotics, while DVT was the most common medical complication noted. The single case of implant dislocation was following THA in a 70-year-old female with an mFI-5 score of 2, who suffered a ground-level fall at home after discharge. Female gender and age >70 years have also previously been identified as risk factors for implant dislocation after THA [16]. Incidence of surgical site infections has been shown to vary from <1% to 3% in international literature, with increased risks associated with the presence of comorbidities, history of smoking, as well as male gender in some studies [15,17].

For the patients undergoing total joint arthroplasty, several authors have used different scores for quantification of the comorbid status. In a study by Rasouli MR et.al, a Charlson Comorbidity Index of ≥ 2 was shown to be significantly associated with the development of surgical site infection with a p-value of 0.01 [17]. In another study by Hustedt JW et.al, in 2017, consisting of a cohort of 4,323,045 patients, a greater number of patient comorbidities was shown to result in increased risk for postoperative complications such as pulmonary embolism, acute myocardial infarction, and respiratory distress syndrome [7]. In 2019, Traven SA et al. used the mFI-5 score to predict complications following THA and TKA and reported an increased number of postoperative complications as well as re-admission and mortality rates with increased mFI-5 scores. They concluded that mFI-5 was an effective and simple score to predict post-operative morbidity and mortality, and should be used to identify surgical candidates with a higher risk of post-surgery morbidity [9]. Similar to these findings, a higher mFI-5 score was also shown to be associated with an increased risk of surgical complications in the current study. This association can be attributed to patient factors such as diabetes and pneumonia, which have been shown to increase the risk of postoperative wound infections (the most common surgical complication encountered in our study) [13,15], and are also components of the mFI-5 score. However, no significant association was observed with respect to medical complications (such as DVT, pulmonary embolism, myocardial infarction), or re-admission and mortality rates, probably due to a small sample size leading to low frequency of observed complications.

Another significant finding observed in the current study is the association of smoking with post-operative medical complications as well as 30-day mortality rate. In a systematic review conducted in 2019, smoking was shown to have a significant association with the development of cardiac complications [5]. In another national database study, smoking was found to be a significant risk factor for 30-day readmission and return to the operating room [13]. Similarly, in another cohort study including a total of 117,024 patients, smoking was shown to increase the risk of medical complications (e.g. acute myocardial infarction and pulmonary infections) and one-year mortality after total joint arthroplasty [18]. Out of the two mortalities reported in the current study, one of the patients developed bilateral pulmonary embolism while the other had a sudden cardiopulmonary arrest, and smoking was found to be a significant risk factor. Based on these findings, it is recommended to consider the risk vs benefit of arthroplasty amongst smokers and counsel them accordingly. Although there is limited literature to emphasize any significant importance of smoking cessation pre-operatively, studies have shown a positive effect on postoperative morbidity following cessation four to eight weeks pre-operatively [18-20].

It is important to note that all five patients requiring re-admission within 30 days were those following THA. One patient with implant dislocation underwent closed reduction and was managed conservatively, while two patients were admitted for surgical site infection and discharged on intravenous antibiotics. Another patient had suffered a bilateral pulmonary embolism and subsequently expired, whereas one of the patients presented with another episode of fall resulting in a contralateral neck of femur fracture. Similar to these findings, a recent study conducted in 2020 also showed a higher re-admission rate following THA vs TKA (3.4% vs 2.2%) [21]. Additionally, the most common reasons for re-admissions were also identified to be infection followed by a history of trauma in the THA group [21].

The results of this study successfully identify the mFI-5 score as a predictor for postoperative surgical complications in our population, similar to the findings in the internationally reported literature. Furthermore, smoking is also shown to be associated with a higher risk of postoperative morbidity and mortality, contributing to the previously available data. These findings highlight the importance of considering patient co-morbidities and smoking status in pre-operative counseling and utilizing these details in predicting the risk of postoperative complications. However, due to the low incidence of systemic complications and other outcome measures, we were unable to report any additional associations, which otherwise may be of clinical importance. Therefore, further multicenter studies with larger sample sizes are still required to reach definitive conclusions.

## Conclusions

TJA has been associated with multiple postoperative complications, with comorbid status proven to have a significant association. The results of the current study show that the mFI-5 index can effectively be used as a predictor of postoperative complications in our part of the world and should be calculated routinely. This score can therefore aid in pre-operative assessment as well as patient counselling.

## References

[1] Fransen M, Bridgett L, March L, Hoy D, Penserga E, Brooks P (2011). The epidemiology of osteoarthritis in Asia. Int J Rheum Dis.

[2] Colby SL, Ortman JM (2015). Projections of the size and composition of the US population: 2014 to 2060. Curr Popul Rep.

[3] Ethgen O, Bruyère O, Richy F, Dardennes C, Reginster JY (2004). Health-related quality of life in total hip and total knee arthroplasty. A qualitative and systematic review of the literature. J Bone Joint Surg Am.

[4] Bannister G, Ahmed M, Bannister M, Bray R, Dillon P, Eastaugh-Waring S (2010). Early complications of total hip and knee replacement: a comparison of outcomes in a regional orthopaedic hospital and two independent treatment centres. Ann R Coll Surg Engl.

[5] Elsiwy Y, Jovanovic I, Doma K, Hazratwala K, Letson H (2019). Risk factors associated with cardiac complication after total joint arthroplasty of the hip and knee: a systematic review. J Orthop Surg Res.

[6] Kurtz SM, Lau EC, Ong KL, Adler EM, Kolisek FR, Manley MT (2017). Which clinical and patient factors influence the national economic burden of hospital readmissions after total joint arthroplasty?. Clin Orthop Relat Res.

[7] Hustedt JW, Goltzer O, Bohl DD, Fraser JF, Lara NJ, Spangehl MJ (2017). Calculating the cost and risk of comorbidities in total joint arthroplasty in the United States. J Arthroplasty.

[8] Ondeck NT, Bovonratwet P, Ibe IK (2018). Discriminative ability for adverse outcomes after surgical management of hip fractures: a comparison of the Charlson comorbidity index, Elixhauser comorbidity measure, and modified frailty index. J Orthop Trauma.

[9] Traven SA, Reeves RA, Sekar MG, Slone HS, Walton ZJ (2019). New 5-factor modified frailty index predicts morbidity and mortality in primary hip and knee arthroplasty. J Arthroplasty.

[10] McDowell I, Hill G, Lindsay J (2001). An overview of the Canadian study of health and aging. Int Psychogeriatr.

[11] Velanovich V, Antoine H, Swartz A, Peters D, Rubinfeld I (2013). Accumulating deficits model of frailty and postoperative mortality and morbidity: its application to a national database. J Surg Res.

[12] Teo BJ, Yeo W, Chong HC, Tan AH (2018). Surgical site infection after primary total knee arthroplasty is associated with a longer duration of surgery. J Orthop Surg (Hong Kong).

[13] Courtney PM, Boniello AJ, Berger RA (2017). Complications following outpatient total joint arthroplasty: an analysis of a national database. J Arthroplasty.

[14] Pulido L, Parvizi J, Macgibeny M, Sharkey PF, Purtill JJ, Rothman RH, Hozack WJ (2008). In hospital complications after total joint arthroplasty. J Arthroplasty.

[15] Mistry JB, Naqvi A, Chughtai M (2017). Decreasing the incidence of surgical-site infections after total joint arthroplasty. Am J Orthop Belle Mead NJ.

[16] Brooks PJ (2013). Dislocation following total hip replacement: causes and cures. Bone Joint J.

[17] Rasouli MR, Restrepo C, Maltenfort MG, Purtill JJ, Parvizi J (2014). Risk factors for surgical site infection following total joint arthroplasty. J Bone Joint Surg Am.

[18] Matharu GS, Mouchti S, Twigg S, Delmestri A, Murray DW, Judge A, Pandit HG (2019). The effect of smoking on outcomes following primary total hip and knee arthroplasty: a population-based cohort study of 117,024 patients. Acta Orthop.

[19] Møller AM, Villebro N, Pedersen T, Tønnesen H (2002). Effect of preoperative smoking intervention on postoperative complications: a randomised clinical trial. Lancet Lond Engl.

[20] Thomsen T, Villebro N, Møller AM (2014). Interventions for preoperative smoking cessation. Cochrane Database Syst Rev.

[21] Phruetthiphat OA, Otero JE, Zampogna B, Vasta S, Gao Y, Callaghan JJ (2020). Predictors for readmission following primary total hip and total knee arthroplasty. J Orthop Surg (Hong Kong).

